# MicroRNA-143 Downregulates Interleukin-13 Receptor Alpha1 in Human Mast Cells

**DOI:** 10.3390/ijms140816958

**Published:** 2013-08-19

**Authors:** Shaoqing Yu, Ruxin Zhang, Chunshen Zhu, Jianqiu Cheng, Hong Wang, Jing Wu

**Affiliations:** 1 Department of Otolaryngology, Huadong Hospital, Fudan University, Shanghai 200040, China; E-Mails: yu_shaoqing@163.com (S.Y.); whhb1984@163.com (H.W.); dushixingniu@163.com (J.W.); 2 Department of Otolaryngology, Tongji Hospital, Tongji University, Shanghai 200065, China; 3 Department of Otolaryngology, Jinan General Hospital of PLA, Jinan 250031, Shandong, China; E-Mails: zhucs1@sina.cn (C.Z.); showing100@163.com (J.C.)

**Keywords:** microRNA-143, IL-13Rα1, mast cell, lentiviral vector, allergy

## Abstract

MicroRNA-143 (miR-143) was found to be downregulated in allergic rhinitis, and bioinformatics analysis predicted that IL-13Rα1 was a target gene of miR-143. To understand the molecular mechanisms of miR-143 involved in the pathogenesis of allergic inflammation, recombinant miR-143 plasmid vectors were constructed, and human mast cell-1(HMC-1) cells which play a central role in the allergic response were used for study. The plasmids were transfected into HMC-1 cells using a lentiviral vector. Expression of IL-13Rα1 mRNA was then detected by reverse transcriptase polymerase chain reaction (RT-PCR) and Western Blotting. The miR-143 lentiviral vector was successfully stably transfected in HMC-1 cells for target gene expression. Compared to the control, the target gene IL-13Rα1 was less expressed in HMC-1 transfected with miR-143 as determined by RT-PCR and Western Blotting (*p* < 0.05); this difference in expression was statistically significant and the inhibition efficiency was 71%. It indicates that miR-143 directly targets IL-13Rα1 and suppresses IL-13Rα1 expression in HMC-1 cells. Therefore, miR-143 may be associated with allergic reaction in human mast cells.

## 1. Introduction

Allergy is a chronic inflammatory condition and many inflammatory factors, such as IL-4, IL-13, IL-8, *etc*. have been related to this disorder [1,2]; however, how these factors regulate mechanisms responsible for allergic reactions has not yet been thoroughly elucidated. Recently, it has been found that microRNAs (miRNAs) inhibit protein translation to regulate gene expression; miRAs are small RNAs produced by cells through a unique process, involving RNA polymerase II, microprocessor protein complex, and the RNAase III/Dicer endonuclease complex. miRNA ribonucleoprotein complex is formed at the end of this process [3]. The biological functions of miRNAs depend on their ability to silence gene expression, primarily through degradation of the target mRNA and/or translational suppression mediated by the RNA-induced silencing complex (RISC) [3]. The function of miRNAs in gene regulation has been investigated in a variety of diseases including allergic disease.

In our previous study, miRNA profiles and RT-PCR were used to reveal the difference between nasal mucosa biopsies of patients with allergic rhinitis (AR) and those of healthy volunteers. When compared with normal tissue, microRNA-143 (miR-143) was the most significantly downregulated miRNA in nasal mucosa of tissues exhibiting AR. It was found that expression of miR-143 in smooth muscle cells of airways triggers allergic conditions such as asthma [4]. However, we have not yet been able to understand how miR-143 regulates allergic inflammation in upper airways such as AR.

To study the mechanism of miR-143 regulation in allergic inflammation, we investigated the target genes of miR-143 through the TargetScan procedure (http://genes.mit.edu/tscan/targetscanS.html and http://pictar.mdc-berlin.de/) to predict miR-143 target genes, and found that the IL-13Rα1 gene 3′ UTR had 15 sequential pairing bases with miR-143 (Table 1), which indicates that IL-13Rα1, known to play an important role in allergy, may be a potential target of miR-143. To investigate this, we used mast cells, which are an important AR target cell and built the miR-143 overexpression system. This system was transfected into mast cells to observe changes induced through target gene expression. The aim of this study was thus to identify the effects of miR-143 on IL-13Rα1 in mast cells to further an understanding of the molecular mechanisms of allergic inflammation.

## 2. Results

### 2.1. Construction and Identification of miR-143 pLenO-RIP Plasmid

**Figure 1:**
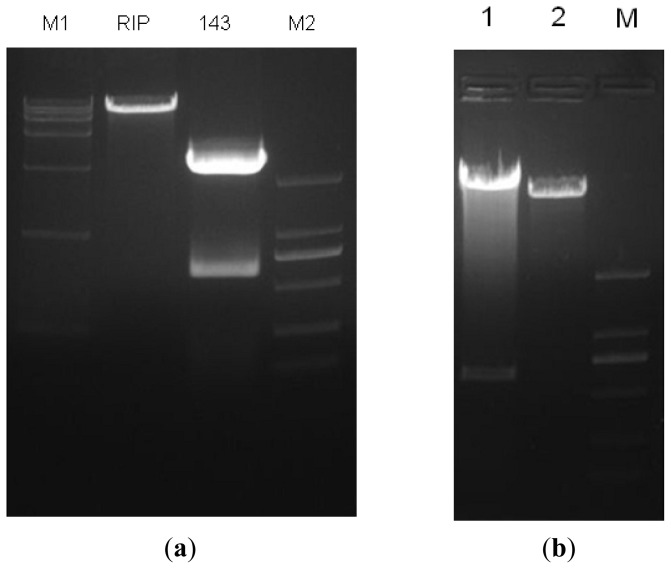
Images of gel of plasmid digest of pLenO-RIP, PUC-miR-143 and recombinant plasmid. Lanes 1–4 (from left to right) were products of DNA marker (DL15000), pLenO-RIP, PUC-miR-143 and DNA marker (DL2000), respectively (**a**). From left to right were positive clones, negative control and marker (DL2000) respectively. The approximate 600bp size of the product can be found in positive clones (**b**).

We connected the target gene DNA fragment into pLenO-RIP empty vector (Figure 1a), and transformed the plasmid ligation product into *E. coli* DH5. Subsequently, we extracted the plasmids from amplified DH5. An approximately 600 bp DNA fragment was identified from the recombinant plasmid (Figure 1b). Finally, DNA sequencing confirmed the recombinant plasmid DNA sequence.

### 2.2. Lentiviral Vector Packaging and Identification

**Figure 2:**
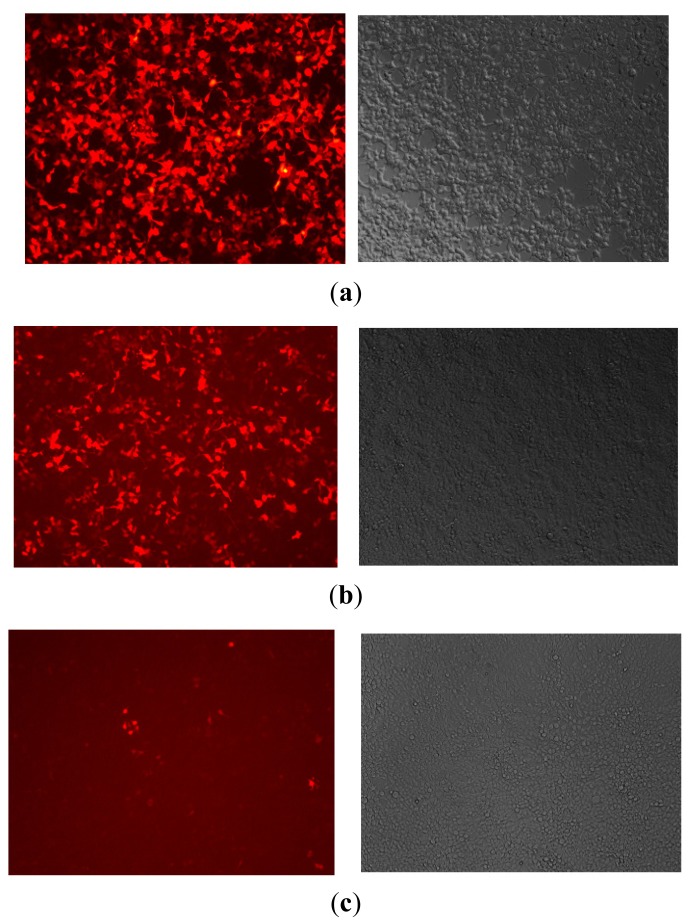
Different concentrations of virus were co-transfected into 293T cells: 0.1 μL (**a**), 0.1 μL (**b**) and 0.01 μL (**c**). Cells grew well and strong fluorescence intensity was observed under the fluorescent microscope, suggesting that the virus packaging was successful.

The lentiviral packaging systems, comprising four kinds of plasmid DNA solutions, were co-transfected into 293T cells. Cells grew well and strong fluorescence intensity was observed under the fluorescent microscope, indicating that the virus packaging was successful (Figure 2). Forty-eight hours after transfection, supernatant viral material was collected and concentrated, then a 10-fold dilution was transformed into a 100–10^−5^ concentration gradient to infect 293T cells, and the supernatant viral material was collected. 48 h later, the virus titer was determined via the virus titer formula (the Tu/mL) = (GFP − positive cell count × virus supernatant dilution factor)/0.1. The production of virus droplets was 2.9 × 10^8^ of TU/mL, and they were then prepared for transfection of HMC-1 cells.

**Figure 3:**
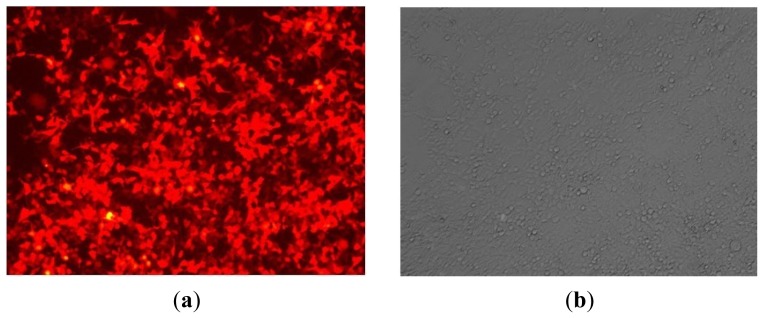
miR-143 transfected HMC-1 cells (**a**) fluorescent vision and (**b**) normal vision. 36 h after transfection, the RFP red fluorescent protein gene were seen expressed in up to 80% cells under fluorescent vision, it showed that the target genes were successfully expressed in the cells. (original magnification ×40)

Forty-eight hours after developing HMC-1 cell culture, the lentiviral vector of miR-143 was transfected into cell culture, and RFP blank lentivirus was used as the control. Approximately 48 h–72 h after infection, the cell culture was found in good condition under an inverted fluorescence microscope, wherein the infection efficiency was up to 80% (Figure 3). The results indicate that the miR-143 lentiviral vector was successfully transfected into HMC-1 cells, and that it expressed the relevant target genes in a stable manner.

### 2.3. miR-143 Suppresses IL-13Rα1 Expression in HMC-1 Cells

**Figure 4:**
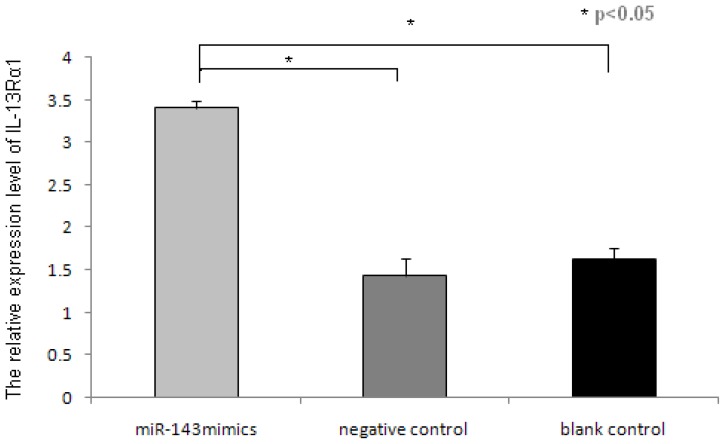
Expression of target gene IL-13Rα1 in different groups by RT-PCR. For the three groups: miR-143 mimics transfected cells, negative control group (empty vector transfected cells) and blank control group (HMC-1 cells), and every experiment was conducted in triplicate. The vertical axis represents the relative expression level of IL-13Rα1 control to 18sRNA (ΔCt). With the same cycle number, among them, miR-143 transfected cells had less amplification of IL-13Rα1 control to 18sRNA (*p < 0.05 for each).

The cumulative data for mRNA expression of IL-13Rα1 are presented in Figure 4. Compared with the control, IL-13Rα1 mRNA expression was significantly downregulated in miR-143 transfected cells (*p* > 0.05). No significant changes of IL-13Rα1 mRNA levels were observed in HMC-1 cells and empty vector transfected cells (*p* > 0.05).

As shown in Figure 4, the expression of IL-13Rα1 in the miR-143 transfected HMC-1 cells group was reported to be 3.41; this value is relative to the internal reference 18sRNA. Furthermore, the expression of IL-13Rα1 in untreated controls HMC-1 and empty vector transfected control group were 1.63 and 1.43, respectively. The results indicated there was significantly reduced expression of IL-13Rα1 when miR-143 is overexpressed in HMC-1 cells, with an inhibition efficiency of 71%. This indicates that IL-13Rα1 is a target gene of miR-143 in HMC-1 cells, and that miR-143 can significantly inhibit the target gene IL-13Rα1 expression in HMC-1 cells.

### 2.4. Western Blotting Results of IL-13Rα1 Downregulation in miR-143 Transfected HMC-1 Cells

**Figure 5:**
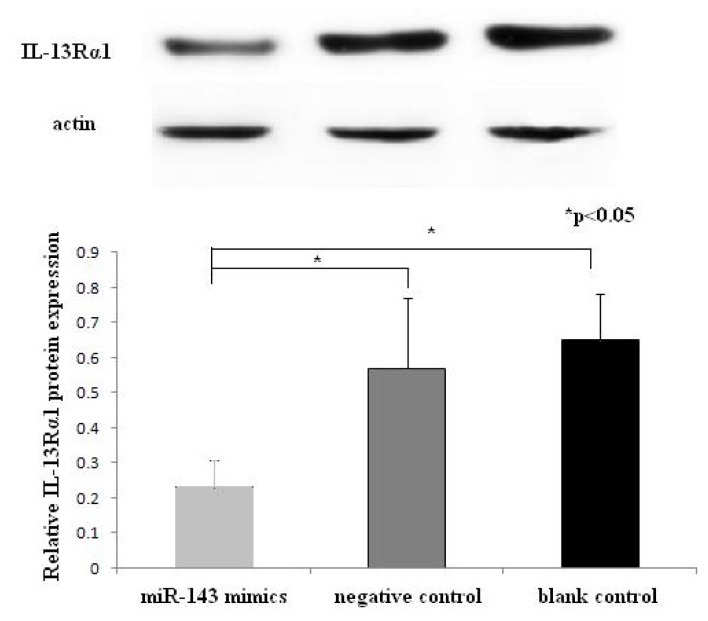
Western blot analysis of IL-13Rα1 protein 48 h after transfection. For the three groups: miR-143 mimics, negative control group and blank control group. Experiments were done in duplicate. Western blot analysis showed that the relative expression of IL-13Rα1 protein in the miR-143 group was much lower than in negative and blank control groups (*p < 0.05 for each).

Western blot analysis showed that the expression of IL-13Rα1 protein in the miR-143 group was much lower than in the negative and blank control groups (*p* < 0.05 for each). There was no significant difference in the expression of IL-13Rα1 protein between the negative control and blank control group (*p* > 0.05). As shown in Figure 5, the over-expression of miR-143 caused reduction in IL-13Rα1 protein expression in HMC-1 cells.

## 3. Discussion

MiRNAs are short (20–24 nt), non-coding RNAs that are involved in post-transcriptional regulation of gene expression in multicellular organisms, because they affect both the stability and translation of mRNAs [5]. In mammals, thousands of miRNAs that perform diverse functions have been identified. Among them, miR-143 was found to be associated with allergic rhinitis in our research [6], but the mechanism of its action was unclear. In order to study this mechanism, we determined the target genes of miR-143 and investigated related mechanisms. The target genes of miR-143 were screened using miRanda (http://www.microrna.org) software. It was found that miR-143 may target multiple genes, including NOVA1, ZIC3 and MARCKS etc. Among these target genes, IL-13α1 has been the focus of allergic inflammation-associated research in recent years [7]. miR-143 caused allergic inflammation, which might be associated with the target gene IL-13α1. Therefore, we carried out this microRNA intervention project.

To determine the gene regulation of miRNA, methods of overexpression and suppression of miRNAs are often used in research studies focused on this subject [8]. In this study, the overexpression of miR-143 was chosen for exploring the regulation of miR-143 on genes of inflammatory cytokines in allergic inflammation. The precursor sequence of miR-143 was synthesized directly, and then this sequence amplified genes using the primer extension method. The product was digested and inserted into the lentivirus expression vector; thus lentiviral packaging plasmids were used to transfect 293T cells. The results indicated that the miR-143 lentiviral expression vector was successfully built which could be perfomed for the next step.

Lentiviral transduction vectora were used in this study because they have the ability to infect different types of cells. In addition, lentiviral transduction vectors can also carry exogenous genes and integrate them into the genome of the host cell for the purpose of achieving long-term stable expression [9]. It will not encode viral proteins, so it can be used for miRNA overexpression transfection experiments [10]. The viral vector carrying a red fluorescent protein gene RFP can be transfected into HMC-1 by lentiviral vectors [11]. The high expression of RFP indicates the high efficiency of miR-143 transfection. Moreover, the sustained expression of RFP in HMC-1 also showed that there was long-term overexpression of miR-143 in cells.

It has been reported that miR-143 plays an important role in regulating smooth muscle cell (SMC) fate and plasticity. Thomas Boettger’s study revealed that the expression of miR-143, which formed a small cluster on mouse chromosome 18, strongly correlated with the number of SMCs. Vascular smooth muscle cells (VSMCs) from miR-143-deficient mice were locked in the synthetic state, which incapacitated their contractile abilities and favored neo-intimal lesion development, thereby revealing an unanticipated role of miR-143 in the regulation of VSMC phenotype [12]. Pleiotropic cytokines, IFN-beta and IFN-gamma, could stimulate miR-143 expression of smooth muscle cell in airways, contributing to airway allergic diseases such as asthma [13]. Some studies also demonstrated that miR-143 deficiency is associated not only with altered vasocontraction but also with impaired vasodilation [14]. Furthermore, some authors showed that miR-143 is a critical regulator of cell cycle activity in stem cells [15]. Recently it was discovered that miR-143 has tumor suppressor activity [16], but the role of miR-143 in allergic inflammation, especially in functional cells, such as mast cell, EOS, and T cell has been little investigated. This is the first study to illustrate that the allergic reaction in mast cells was attributable to inhibition of IL-13Rα1 by miR-143.

IL-13 is an immunoregulatory cytokine predominantly secreted by activated Th2 cells. Over the past several years, it has become evident that IL-13 is a key mediator in the pathogenesis of allergic inflammation. Like IL-4, IL-13 responds by signaling through the T-cell antigen receptor and mast cells. IL-13 responds through basophils when there is a cross-linkage of the high-affinity receptor for IgE. It is also produced by activated eosinophils. IL-13 plays a pivotal role in IgE-dependent inflammatory reactions, and it acts on B cells to produce IgE. IL-13 has been implicated in a variety of allergic responses, including airway hypersensitivity, mucus hypersecretion, AR, and asthma. IL-13 induces many of the same responses as IL-4 and shares a receptor subunit with IL-4 [17,18].

IL-13 mediates its effects by interacting with a complex receptor system comprised of IL-4Rα and two IL-13 binding proteins: IL-13Rα1 and IL-13Rα2. The expression of IL-13 receptors has been detected on human B cells, basophils, eosinophils, mast cells, endothelial cells, fibroblasts, monocytes, macrophages, respiratory epithelial cells, and smooth muscle cells. Human IL-13Rα1 is a single gene on chromosome Xq13 [19]. It binds IL-13 with low affinity, and also combines with IL-4Rα to form a high-affinity IL-13 binding complex. A variety of cells, including B cells, basophils, eosinophils, mast cells, endothelial cells, fibroblasts, monocytes, epithelial cells, and smooth muscle cells, respond to IL-13 owing to this binding complex.

Some studies have shown that human mast cell can express IL-13Rα1 and can be activated by IL-13 [20]. In this study, the overexpression of miR-143 can suppress the expression of IL-13Rα1 in mast cells, which may be stimulated by combined IL-13/IL-4. This ultimately translates into minimizing the allergic response. Thus, the role of miR-143 in allergic response may be associated with regulation of IL-13 pathway. Nevertheless, further investigation is needed to validate these inferences.

## 4. Experimental Section

### 4.1. Construction of miR-143 Target Sequence-Luciferase Reporter Plasmid

**Figure 6:**
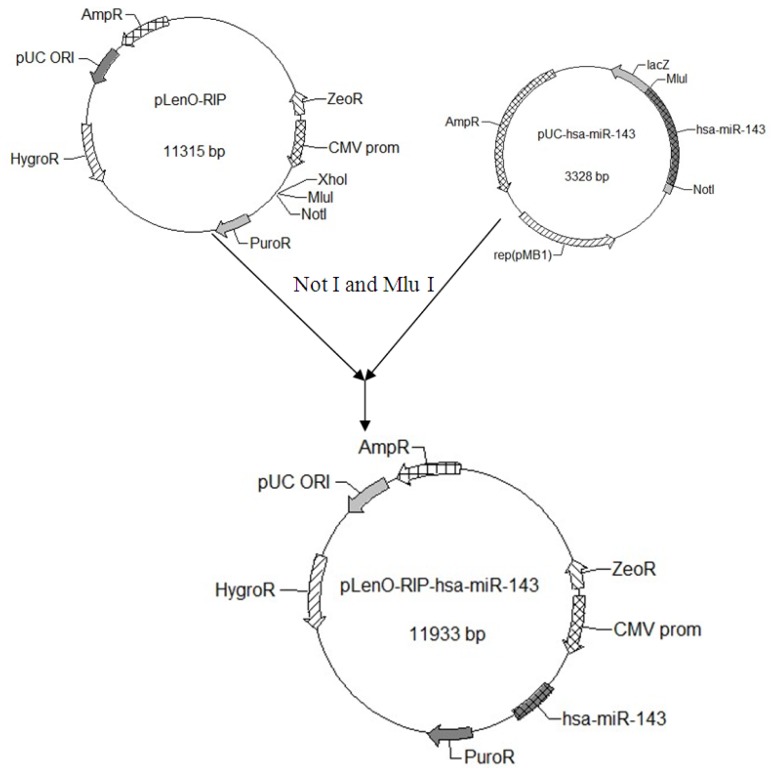
Construction of miR-143 target sequence-luciferase reporter plasmid.

Using the miR-143 target sequence obtained from Ensembl, we designed its pre-miRNA sequence (GCGCAGCGCCCUGUCUCCCAGCCUGAGGUGCAGUGCUGCAUCUCUGGUCAGUUGGGAG UCUGAGAUGAAGCACUGUAGCUCAGGAAGAGAGAAGUUGUUCUGCAGC). We extended both sides of the sequence of pre-miRNA about 200 bp as the pri-miRNA. The sequence with an over-hanging Mlu I site and a Not I site was synthesized by Genscript (Shanghai, China) and digested to obtain the target gene fragment. The miR 143-coding genomic DNA fragment was cloned down stream from the RFP gene of the lentiviral vector pLenO-RIP by digesting with Not I and Mlu I (Figure 6). The constructs were confirmed by DNA sequencing.

### 4.2. Lentivirus Production and Transfection

Lentivirus vector was used to transfect genes. This virus packaging system was composed of pRsv-REV, pMDlg-pRRE pMD2G, and interference plasmid. With the help of Lectivirus vector, we could determine the gene expression plasmid, the (pLenO-RIP + miR-143), which also containing the red fluorescent protein (RFP). The production of lentiviral vector pLenO-RIP + miR-143 was performed by simultaneously delivering lentiviral transfer vectors and packaging plasmids (pRsv-REV, pMDlg-pRRE and pMD2.G) into 293 T cells.

Pseudo-viral particles that were generated by 293 T cells within 48 h were centrifuged at 100,000× *g* for 2 h and frozen at −70 °C for future experiments. 293 T cells were seeded into 24-well plates, and lentivirus was used to transduce for 48 h with a multiplicity of infection (MOI) of 10.

### 4.3. Cell Culture

The HMC-1 cells were cultured at 37 °C in an atmosphere of 5% CO_2_. In this case, HMC-1 cells were cultured in DMEM media supplemented with 10% FBS, and the media were replaced routinely. The cells were subjected to transfection when the cell density was about 60%–70%.

### 4.4. Transfection and Luciferase Assays

To eliminate the interference of transfection reagent, cell lines were seeded into 24-well plates and set up as follows: (a) miR-143 mimics group, in which the cells were transfected with lentivirus at a multiplicity of infection (MOI) of 10 for 48 h; (b) negative control group, in which the cells were subjected to transfection with lentiviral without miR-143; and (c) blank control group, in which the HMC-1 cells were cultured routinely without any treatment. All cells of groups were cultured in normal conditions. The cells were subjected to transfection when the cell density was about 60%–70%. All transfections were performed in quadruplicate and repeated in at least three independent experiments.

After transfection, cell count was determined at 24 h, 48 h and 72 h, respectively for the purpose of determining cell proliferation. The expression of RFP was observed as red fluorescent protein under fluorescent microscope. Moreover, RNA and protein were extracted after transfection at 48 h or 72 h, respectively.

### 4.5. RNA Isolation and Reverse Transcription and Polymerase Chain Reaction (RT-PCR) for miR-143-Target Gene Expression

Total cellular RNA was extracted from 1 × 10^6^ cells using TrizolTM reagent (Invitrogen Inc., Carlsbad, California, MD, USA) according to the manufacturer’s protocol. The concentration and purity of RNA were determined spectrophotometrically. Then, the synthesis of cDNA was performed according to a cDNA cycle kit K1621 (Fermentas Inc., Burlington Canada). To determine the expression of the target gene of miR-143, IL-13Rα1, we performed fluorescent quantitative real time RT-PCR assay. The sequences of the primers (TaKaRa Inc., Dalian, China) specific for IL-13Rα1 were performed with sense (ACCCGAGGGAGCCAGCTCAA) and antisense (GGTGCTACACTGGGACCCCACT) primers, wherein the expected size of the amplified sequence was 111 bp. 18sRNA was used as control. Then, the incubation of cDNA and primer was performed at 95 °C for 15 s, and the PCR reaction proceeded for 45 cycles as per the following conditions: 95 °C for 15 s and 60 °C for 30 s, in a programmable thermal cycler (Eppendorf realplex.2s, Eppendorf Co., Ltd., Hamburger, Germany) using a thermostable Taq DNA polymerase (SYBR premix ex taq, TaKaRa Inc., Dalian, China). Experiments were done in triplicate. For each sample, the amounts of the target and an endogenous control (18sRNA) were determined. To obtain a normalized target value, the amount of the target molecule was divided by the amount of the endogenous reference.

### 4.6. Western Blotting Analysis

Forty-eight hours after transfection, HMC-1 cells were harvested and centrifuged, and total protein was extracted. Protein concentrations were determined using the BCA protein assay. After heated for 10 min at 100 °C, 20 g of denatured protein was subjected to 10% SDS-PAGE. Then proteins were transferred electrophoretically for 1 h at 200 mA at 4 °C onto PVDF membranes. Membranes were blocked for 1 h at room temperature in TBS containing 5% non-fat dry milk. Blots were washed 3 times for 10 min each with 0.1% TBS-T and subsequently treated overnight at 4 °C with primary antibodies against IL-13Rα1 and actin (1:500). After washing 3 times for 10 min each with 0.1% TBS-T, the blots were incubated with anti-mouse antibody (1:5000) conjugated with horseradish peroxidase for 1 h at room temperature. Bands were visualized by using EZ-ECL detection reagents. The scanned images were quantified using Quantity One software. Actin used as an endogenous protein for normalization. Experiments were done in duplicate. The ratio of IL-13Rα1/actin was used for semi-quantification and comparison between different groups.

### 4.7. Statistical Analysis

All data were analyzed by SPSS10.0 software (SPSS, Chicago, IL, USA, 2000). Microarray data were analyzed by Cluster Analysis. All data were represented by mean values ± standard deviation. P values less than 0.05 were considered statistically significant. Independent simple T test was used to compare the results of different groups.

## 5. Conclusions

We successfully constructed the lentiviral miR-143 overexpression vector and transfected it into human mast cell. In this study, we also identified IL-13Rα1 as a target gene of miR-143, and that it was inhibited by overexpression of miR-143 in mast cells. All these findings indicate that miR-143 may play important roles in triggering allergic inflammation.

## Figures and Tables

**Table 1:** Target prediction of IL-13Rα1 for Has-miR-143 based on conservation.

Nuclei mapped to alignments	Nuclei mapped to sequence	Structure of predicted duplex	Probabilities	Free energies kcal/mol
1397	1338	_GAG__GCAG__G________CAUCUCA_: _CUC__UGUC__C________GUAGAGU_	0.30	−24.1
